# Harnessing microbiome‐based biotechnologies for sustainable mitigation of nitrous oxide emissions

**DOI:** 10.1111/1751-7915.12758

**Published:** 2017-07-11

**Authors:** Hang‐Wei Hu, Ji‐Zheng He, Brajesh K. Singh

**Affiliations:** ^1^ Faculty of Veterinary and Agricultural Sciences The University of Melbourne Parkville VIC 3010 Australia; ^2^ Hawkesbury Institute for the Environment Western Sydney University Penrith NSW 2751 Australia; ^3^ Global Centre for Land‐Based Innovation Western Sydney University Penrith NSW 2751 Australia

## Abstract

Achieving the Sustainable Development Goal of climate change mitigation within this century will require adoption of new innovative technologies to control emissions of nitrous oxide (N_2_O), an important greenhouse gas leading to global warming. This is particularly important in the face of growing fertilizer consumption and continuous land degradation. Currently used tools to mitigate N_2_O emissions are based on agrochemical inputs and agronomic practices. Emerging technologies include plant breeding approaches to manipulate microbiome activities in agro‐ecosystems, and microbial biotechnology approaches for *in situ* microbiome manipulation and engineering via use of biochemical, cellular and genome‐editing methods. This article assessed the likely contribution of microbial biotechnology to the mitigation of N_2_O emissions and discussed how to facilitate the development of environmental‐friendly microbiome‐based biotechnology for sustainable climate change mitigation.

## Global climate change and nitrous oxide emissions

A key component of the Sustainable Development Goals (SDG) formulated by the United Nations on 25th September 2015 is to ‘take urgent action to combat climate change and its impacts’ (SDG goal 13). Given current greenhouse gas (GHG) concentrations and projected emissions, global temperatures will likely increase by 1.2–4.8°C within this century. To keep the increase in temperature <2°C is a global challenge which necessitates reducing GHG emissions without compromising food security for increasing global population under increasing frequencies and intensity of extreme weather events. Nitrous oxide (N_2_O) is the third most important GHG and an ozone‐depleting substance. Its concentration has substantially increased from preindustrial levels of 270 ppb to current levels of 324 ppb, and global N_2_O emission is projected to further increase by 35–60% before 2030, owing to the increasing application of nitrogen (N) fertilizers in agriculture, which contributes 59% of total N_2_O emissions. Over the past several decades, extensive studies have resulted in a growing understanding on mechanisms that underpin microbial N_2_O production and consumption processes through multiple biological pathways (Singh *et al*., 2010; Hu *et al*., 2015). As a consequence, mitigation tools developed and utilized are focused towards either manipulation of abundance/structure/activities of N_2_O‐relevant microbiomes or reducing the amount of N resources available to microbial N_2_O formation (Hu *et al*., 2017).

## Currently used approaches to mitigate N_2_O emissions

**Figure 1:**
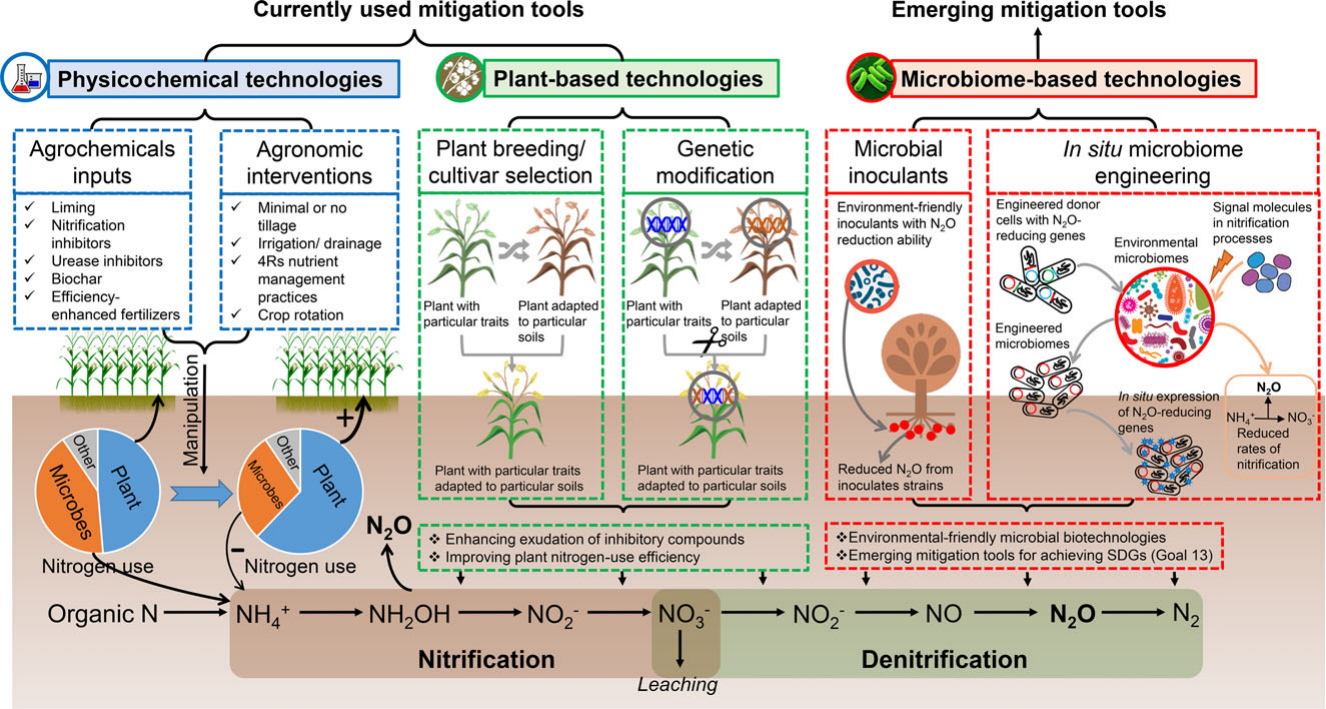
Toolbox for currently used N_2_O mitigation strategies based on physicochemical and plant community technologies, and emerging N_2_O mitigation strategies based on microbiome biotechnologies.

Physicochemical approaches are mostly used as practical tools to eliminate N_2_O formation or to promote its conversion to N_2_ in agro‐ecosystems (Fig. 1). Some strategies include manipulation of soil biotic and abiotic properties (e.g. soil pH, carbon: nitrogen ratio, moisture and cover crops) by agrochemical amendments and agronomic practices, utilizing high‐efficiency fertilizers (e.g. fertilizers with surface coatings to control release of nutrients), use of urease and nitrification inhibitors (Shi *et al*., 2016) and 4Rs (right source, right rate, right time and right place) nutrient management practice for synchronizing N supply and crop N demand. However, these approaches cannot consistently reduce N_2_O emissions across various field conditions without affecting other biological N cycling pathways. Some agronomic practices have proved to induce considerable shifts in taxonomic and functional traits of soil microbiomes, which would impact ecosystem functioning worldwide including food production by impacting nutrient cycling (Leff *et al*., 2015). Long‐term repetitive use of agrochemicals, in particular, has negative biological and environmental impacts, resulting in the accumulation of undesired residues in fields and food, loss of beneficial microbes and impacts on the evolutionary association between plant and soil microbiota (Singh and Trivedi, 2017).

Plant‐based mitigation approaches are targeting at improving crop N‐use efficiency (thus reducing N accessible to microbiomes) through plant breeding (cultivar selection) or genetic engineering techniques, and utilizing plant physiological traits such as plant‐exuded nitrification/denitrification inhibitors or specialized signalling molecules for the selection/inhibition of a specific microbiome that benefits reduced N_2_O formation and enhanced N_2_O reduction (Fig. 1). Conventional plant breeding techniques, however, does not have a system‐level view of the relationships among plants (phyllosphere, endosphere, spermosphere and rhizosphere), their environment and interacting microbiomes (bacteria, fungi and viruses), collectively termed as the phytobiome (Leach *et al*., 2017). This may result in disruption of the plant–environment–microbiome interactions and unintended consequences for other ecosystem functions (Singh and Trivedi, 2017). Although some plant species (e.g*. Brachiaria humidicola* and *Fallopia* spp.) can exudate organic compounds that could inhibit the growth of nitrifying/denitrifying microbes (Bardon *et al*., 2014), their ability to mitigate N_2_O emissions was restricted in particular scenarios, especially if the plants are adapted to specific climate and soil types. The creation of genetically modified plants with a greater capacity to synthesize exudates (nitrification/denitrification inhibitors) and signalling molecules to favour the establishment of desired microbiomes is an alternative option. Despite these efforts, however, large‐scale plant breeding and genetic modification programmes rarely take into account the plant–microbiome signalling channels. Future plant‐based strategies should integrate the knowledge of the phytobiome into the programme, by which very specific functions (e.g. nitrification/denitrification) are targeted while increasing plant tolerance to pests/diseases and biotic/abiotic stresses.

## Emerging microbial biotechnology approaches to mitigate N_2_O emissions

### Transplantation of microbial cocktails

Continuous land/water/air pollution and increasing demands for clean and healthy environments call for innovative and sustainable solutions to utilize available natural resources (e.g. soil microbiomes) for climate change mitigation. Microbial biotechnologies have shown great promise in improved N_2_O mitigation effects through the transplantation of foreign microbial cocktails (denitrifiers harbouring N_2_O reductase) into ecologically competitive soil environments (Itakura *et al*., 2013) (Fig. 1). Although cultured microbes possess certain N_2_O reduction capacity, their persistence and functionality after inoculation into a new ecological niche are not clear, as they have often been reported to be outcompeted by indigenous microbiomes or may negatively affect plant and soil health. When using microbial inoculation as a strategy to manipulate agricultural microbiomes, we need to maximize the inoculation success during agronomic practices via developing novel delivery systems. In this regard, new technologies are being developed to overcome the lack of colonization and survival of introduced microbiota in field conditions. These include (i) creating new microhabitats for the introduced microbiota by inducing minor disturbances through the use of chemical pesticides, predators for the indigenous microflora; (ii) use of consortia rather than single species to improve their combined competitive strength; (iii) use of synbiotics (probiotics plus prebiotics, e.g. compost, certain carbon and mineral sources) to provide initial support for colonization by the introduced microbiota (Adam *et al*., 2016); and (iv) use of slow‐release systems (e.g. inocula encapsulated in open‐ended tubes) that provide continual inoculation over considerable periods of time (Boon *et al*., 2002; Mertens *et al*., 2006). However, their efficacy in field conditions still needs to tested and validated. In addition, monitoring the colonization ability of microbial inoculants under a variety of abiotic and biotic conditions will improve our ability to predict their fate and behaviours in agro‐ecosystems.

### Utilizing signalling molecules to manipulate microbiomes

With the advancement of omics‐based technologies (e.g. metagenomics, metatranscriptomics and metaproteomics), we can now explore complex environmental microbiomes in various states and pinpoint specific functional genes and construct many metabolic pathways. Emerging microbial biotechnologies are proposed to precisely manipulate the environmental microbiomes *in situ* over a wide range of magnitudes and specificities (Sheth *et al*., 2016), providing new means to innovate current mitigation tools by reducing agrochemicals inputs while maintaining soil health and mitigation performance under various conditions. These microbial biotechnologies are mostly based on biochemical (e.g. microbial cocktails, signalling molecules), cellular (e.g. probiotics, recombinant communities) and genome‐editing methods (e.g. engineered mobile DNA). Researchers have developed novel means to regulate gene activity in diverse microbiome taxa in the human gut, by simply adding or withdrawing artificial chemicals. New paradigms in medical science can provide approaches to be tested and trialled in agricultural biotechnologies. Various environmental‐friendly products have been available through using agricultural biotechnologies, such as beneficial bacteria to control pests, and signalling molecules to enhance plant–bacteria associations, which has addressed site‐specific needs and enabled high crop productivity. However, these products represent only a minor fraction of the potential benefit that could be provided by fully taking advantage of microbiome knowledge. Multidisciplinary approaches, especially genome engineering and synthetic biology, as well as a system‐level view of interacting microbiome components, are needed for maximizing the contribution of microbiome‐based biotechnologies to sustainably mitigating N_2_O emission from agro‐ecosystems.

A critical step towards a microbiome‐based mitigation solution has been the exploration of the core microbiomes across various environments, and identification of their functional potential for N_2_O production or reduction. These efforts will advance our mechanistic understanding of the microbes involved in nitrification and denitrification (the predominant sources of N_2_O), and yield new intervention points either through direct manipulation of the microbiome or via genetically engineering the native microbiomes *in situ* for reduced rates of nitrification and/or enhanced N_2_O reduction. Microbiome‐based approaches targeting at reducing nitrification rates, in particular, would bring multiple benefits in addition to lowering N_2_O fluxes, including increased farm productivity and food security (SDG goal 2), reduced water contamination by nitrate leaching (SDG goal 6) and higher farm profitability through reducing the use of fertilizers (SDG goal 1). In order to regulate gene activities *in situ* (i.e. inhibiting the activity of N_2_O‐producing microbes, or promoting the activity of N_2_O‐reducing microbes), we need to identify the array of signalling molecules (or their inhibitors) produced or perceived by the interacting microbiomes for chemical communication (Leach *et al*., 2017). It has been reported that quorum sensing signals regulate the cross‐talk between nitrite oxidizers and ammonia oxidizers, and influence production and consumption of N oxide gases (NO, NO_2_ and N_2_O) in a model nitrite oxidizer, *Nitrobacter winogradskyi* (Mellbye *et al*., 2016). These findings have implications for developing artificial chemicals to quench quorum signals in nitrification pathways, or utilize plant traits to secrete compounds to modulate the soil microbiomes mediating N_2_O transformations. Various approaches based on plant–microbe chemical communication have been tested to provide benefits to plants while minimizing input requirements. However, we are just beginning to recognize the specificity and diversity of signalling molecules, and their signalling mechanisms, regulatory frameworks, genomic circuits and cascades of signal transductions remain largely unknown Understanding the composition and significance of these signalling molecules among microbiomes at the ecosystem level, however, is becoming more reliable through integrated metabolome and proteome technologies (Leach *et al*., 2017), which might open up promising microbiome‐engineering strategies that could improve N_2_O mitigation by utilizing naturally evolved microbiome communication channels.

### *In situ* microbiome‐engineering approaches

Apart from mediating N_2_O production/reduction through harnessing signalling mechanisms, it is becoming possible to directly engineer the genomes and metabolic pathways of native microbiomes in a predictable manner (Fig. 1). The emerging synthetic biology and genome‐editing tools can engineer mobile genetic elements (MGEs) (e.g. plasmids and transposons), for targeted manipulation of the native microbiomes for N_2_O mitigation, by modifying functional genes mediating nitrification and denitrification processes. The prevalent MGEs in microbial communities and their mediated transfer to broad microbiome phyla are considered a powerful approach to manipulate diverse communities with high efficacy (Sheth *et al*., 2016). After introducing engineered MGEs into the environment, we need significant advances in tools to reliably monitor the engineering outcomes through enabling their efficient delivery, transfer, propagation and appropriate stability in natural settings. A better understanding of the gene regulation frameworks (e.g. programmable transcriptional and post‐transcriptional regulators), coupled with improved modelling techniques to predict the effects of microbiome manipulations *in situ*, will enable strategies to better control engineered functions in complex communities and sustain enhanced N_2_O mitigation in fields.

Microbiome‐based mitigation approaches should work in diverse environmental and climatic conditions with comparable or even better mitigation effects than conventional physicochemical approaches, and should be economically competitive and socially responsible. However, these emerging *in situ* microbiome‐manipulation tools, particularly use of genetically modified organisms (GMOs) in nature, are subjected to regulatory requirements and societal concerns (Singh and Trivedi, 2017). Scientists should strengthen the engagement with policy makers, industry stakeholders, public and private partners, agricultural communities and society to deliver useful knowledge, promote uptake of new microbial biotechnology innovations and ensure successful implementation. With supportive policies, strategic funding investments to support critical research and infrastructure can be prioritized for developing microbiome‐based mitigation approaches and the timely translation of new biotechnologies to the agricultural sector. Investments by national‐level initiatives (e.g. USA National Microbiome Initiative, China Soil Microbiome Initiative) suggest that increasing microbiome‐based knowledge will be available for N_2_O mitigation tool improvement in future. Whether signalling compounds can be used to manipulate environmental microbiomes, or whether *in situ* engineering of indigenous microbiomes should be targeted, will need to be further investigated. Therefore, utilization of established techniques (chemical inputs, agronomic interventions and conventional plant breeding approaches) in combination with biochemical (e.g. microbial cocktails) technologies seems more practical and will be focus of technological refinements in the short to medium term.

## Concluding remarks

Microbial biotechnology offers a new vision for sustainable climate change mitigation, and its enormous potential in minimizing N_2_O emissions could be achieved through new innovations in microbiome technology, improving and translating new microbiome knowledge into long‐term outcomes. Research priorities to achieve this vision are to characterize diverse microbiome components and their interactions, integrate microbiome‐based knowledge of N_2_O sources and sinks, develop system‐level approaches for microbiome analysis and prediction, optimize practical solutions to add to the climate change mitigation toolbox and apply microbiome‐based mitigation tools with next‐generation site‐specific precision agriculture which can sustain enhanced food production while simultaneously decreasing N_2_O emissions (Box 1). This will require conceptual and technological advances in diverse fields of research, including multi‐omics techniques, systems biology, microbial ecology, synthetic biology, data analytics science and precision agriculture. This paradigm shift in climate change mitigation can potentially lead to increased resilience of our agro‐ecosystems to climate extremes and land degradation; improved management to support long‐term soil health and nutrient security; and reduced negative impacts of chemical inputs on the environment.

Box 1 **Research and Innovation priorities to harness microbiomes to reduce N_2_O emissions.**

A core set of research priorities is outlined to specifically accelerate the integration and translation of new agricultural microbiome‐manipulation approaches into practical N_2_O mitigation tools. This will require multifaceted advances in theoretical and experimental approaches, sequencing techniques, standardized protocols, modelling, data analytics, as well as strong collaborative efforts among scientists, engineers, agribusiness professionals and agricultural communities.

(1) Defining the core environmental microbiome components, dynamics, functions, interactions and their signalling mechanisms. We are just beginning to understand the critical components of microbiomes and how they are impacted by habitats, vegetation, climate, environmental perturbation and agronomic practices, as propelled by advances in multi‐omics approaches. Beyond the taxonomic and physiological knowledge of core microbiomes, understanding their ecological and social networks and signal molecules used by microbes to communicate with each other is essential for successfully attempting to manipulate the agricultural microbiomes.

(2) Unravelling the microbiome‐based knowledge of N_2_ O sources and sinks. Earlier efforts were devoted to the discovery and description of taxonomic diversity, while recent studies have emphasized to elucidate the functional attributes of microbiomes. Targeted metagenomics of functional genes and shotgun metagenomics will enable a better insight into the functional potential of microbes, while metatranscriptomics, metaproteomics and metabolomics will decipher the functional community phenotype. These efforts will result in new knowledge of microbial taxa involved in N_2_O transformations, exemplified by the recent discovery of atypical N_2_O reductase proteins through metagenomes (Orellana *et al*., 2014).

(3) Generation of system‐level knowledge and modelling tools for microbiome analysis and prediction. An integrative understanding of how microbiomes modulate N_2_O emissions as a whole will generate system‐level knowledge that can greatly enhance our capacity to harness these microbiome components to optimize climate change mitigation tools. We need conceptual and predictive models that can integrate the various components of microbiome datasets (including genes, transcripts, proteins and metabolites), soil parameters, weather data across various spatial and temporal scales, which will be combined with climate modelling and new computational methods (e.g. big data analytics technologies).

(4) Development of practical microbiome‐based solutions to enhance climate change mitigation. A comprehensive knowledge base of microbiomes might enable the development of site‐specific management strategies that are tailored to specific microbiome components in specific environments. These strategies may include the transplantation of microbial inoculants and use of microbial products or signal molecules to modulate the presence or activity of target indigenous microbiomes. Coordinated community efforts will be necessary to consolidate and rapidly translate new microbiome knowledge into practical solutions.

(5) Combining microbiome‐based climate change mitigation solutions with next‐generation precision agriculture. Microbiome data can be integrated into established or new tools being developed for precision agriculture which can generate high‐resolution and high‐accuracy data across broad spatial and temporal scales. The integration of diverse types of data will require standardized data collection procedures, data processing and analysis, as well as computational and statistical tools to maximize the interoperability of experimental data.
